# A factor analysis of the meanings of anorexia nervosa: intrapsychic, relational, and avoidant dimensions and their clinical correlates

**DOI:** 10.1186/s12888-016-0894-6

**Published:** 2016-06-07

**Authors:** Enrica Marzola, Corine Panepinto, Nadia Delsedime, Federico Amianto, Secondo Fassino, Giovanni Abbate-Daga

**Affiliations:** Eating Disorders Center for Treatment and Research, Department of Neuroscience, University of Turin, Turin, Italy

**Keywords:** Anorexia nervosa, Meaning, Adaptive function, Ambivalence, Resistance

## Abstract

**Background:**

Anorexia nervosa (AN) is a difficult to treat disorder characterized by ambivalence towards recovery and high mortality. Eating symptomatology has a sort of adaptive function for those who suffer from AN but no studies have to date investigated the relationship between the reported meanings of AN and patients’ clinical characteristics. Therefore, we aimed to perform a factor analysis of a new measure testing its psychometric properties in order to clarify whether subjective meanings of AN can be related to AN severity, to ascertain if some personality traits correlate with the meanings attributed to AN by patients, and finally to verify to what extent such meanings relate to patients’ duration of both illness and treatment.

**Methods:**

Eighty-one inpatients affected by AN were recruited for this study and clinical data were recorded. Participants were asked to complete a novel instrument, the Meanings of Anorexia Nervosa Questionnaire (MANQ) focused on the measurement of values that patients attribute to AN and other measures as follows: Eating Disorders Inventory-2, Beck Depression Inventory, Temperament and Character Inventory, and Anorexia Nervosa Stages of Change Questionnaire.

**Results:**

As measured by the MANQ, body dissatisfaction, problems of adolescence, and distress at school or work mainly triggered the onset of AN. Balance and self-control were mostly reported as meanings of AN while the most frequent negative effects were: being controlled by the illness, obsessive thoughts about body shape, and feeling alone. Differences were found between diagnostic subtypes. When a factorial analysis was performed, three factors emerged: intrapsychic (e.g., balance/safety, self-control, control/power, way to be valued), relational (e.g., communication, way to be recognized), and avoidant (e.g., the avoidance of negative feelings, emotions, and experiences). These factors correlated with patients’ personality and motivation to treatments but were unrelated to duration of both illness and treatments.

**Conclusions:**

Given the ego-syntonic nature of AN, the understanding of patients’ value of their disorder could be relevant in treatment; moreover, the positive value of AN resulted to be unrelated to the duration of both illness and treatments. Future research is warranted to replicate these findings and test their clinical implications.

**Electronic supplementary material:**

The online version of this article (doi:10.1186/s12888-016-0894-6) contains supplementary material, which is available to authorized users.

## Background

Notwithstanding the increased knowledge achieved in recent years on eating disorders, anorexia nervosa (AN) still represents a difficult to treat disorder. In fact, AN sufferers often refuse treatments [1], show poor compliance with therapy leading to high dropout rates [2], relapse [3], and high mortality [4]. A deeper understanding of those factors underpinning patients’ difficulties with treatments is thus relevant to improve clinicians’ therapeutic approach to this challenging disorder.

Consensus has been reached on the adaptive function of eating symptomatology; in fact, starvation would help patients avoid negative emotionality [5, 6], reinforce their identity [7, 8], and express their distress [9]. Consistent with these lines of research, AN symptoms would assume a “pro-AN” function in turn maintaining the disorder [10].

The aforementioned hypotheses are in line with cognitive-behavioral models of AN maintenance [10–12] as well as with psychodynamic models that assess defense mechanisms, ego-syntonicity, and compensation for eating symptoms [13–17]. Such theoretical models [10, 14] rely on a handful of studies investigating patients’ meaning of AN [7, 12, 18, 19]; moreover, the majority of these papers used either qualitative, descriptive, or phenomenological methods. Using interviews or focus groups these studies identified some meanings that are likely to be attributed to AN by sufferers. For example, Nordbø and coworkers [7] identified eight main constructs for AN encompassing security, avoidance, mental strength, self-confidence, identity, care, communication, and death. Williams & Reid [12] highlighted that ambivalence towards treatment correlates with patients’ description of AN not only as a disease but also as a tool and a way to achieve their own identity. Such ambivalent features have been sometimes described as the “anorexic voice” speaking to patients’ recovery oriented parts [12, 18]. Our group has previously identified difference, company, and identity as adaptive areas of AN; notwithstanding, ambivalence towards the illness and negative sequelae of AN were also reported by sufferers [19]. A review of 24 qualitative studies [8] confirmed the existing findings suggesting that patients highly value their preoccupations with food and weight. Moreover, this paper identified some factors that are linked to the meaning of AN and then grouped them into two meta-categories: need of control and identity [8].

To date, only a dearth of quantitative studies measured pros and cons of AN from a subjective standpoint, for example analyzing letters written by individuals with AN to their own eating disorder [20]. Relatedly, the Pros and Cons Anorexia Scale (P-CAN [21, 22]) and then the Pros and Cons Eating Disorders Scale (P-CED [23]) have been proposed. These instruments confirmed that AN sufferers experience a variety of positive feelings towards their illness like safety, identity, and being special, only to name a few. These elements could underpin patients’ ambivalence towards recovery and become less valuable when patients start to improve their clinical condition [24]. However, several aspects of AN have been so far not in-depth investigated, like the role of avoidance in AN and the relationship between the reported meanings of AN and patients’ clinical characteristics like personality, duration of treatment and, most importantly, duration of illness. Still, we attempted to develop a brief instrument since it can be of help in clinical practice. Therefore, the rationale for conducting this study is grounded on these elements.

Therefore, the overarching aim of this study was two-fold: a) to perform a factor analysis of a new measure testing its reliability and validity; b) to verify the correlations of this measure with eating disorder severity, personality, and duration of illness or duration of treatment.

We hypothesized that the new measure would have reliably captured AN meanings in an easy-to-administer way and that certain subjective meanings of AN could be a constitutive element of illness in turn involved in maintaining the disorder. Therefore, we expected to find a correlation between certain meanings of illness and clinical data (particularly personality) independently of duration of both illness and treatment.

## Methods

### Participants

Eighty-one inpatients with AN were enrolled in this study. All participants were recruited between December 2013 and February 2015 while hospitalized at the ward for Eating Disorders of the “Città della Salute e della Scienza” hospital of the University of Turin, Turin, Italy.

To be eligible to participate in this study patients had to meet DSM-IV-TR [25] criteria for AN, as assessed by an experienced psychiatrist with the Structured Clinical Interview for DSM-IV-TR Axis I Disorders (SCID-I [26]). Other inclusion criteria were: a) female gender, and b) age ranging between 18 and 45 years old. Patients with organic comorbidities were excluded. Overall, 4 individuals refused to take part in this study and 3 patients had to be excluded because of concurrent organic comorbidities.

### Procedure and measures

Within the first week of hospitalization patients were asked to complete the following assessment:

- **Meanings of anorexia nervosa questionnaire (MANQ).** This is a novel instrument (fully available as Additional file 1) specifically designed in order to quantitatively evaluate patients’ meanings of AN. The development of this questionnaire was grounded on the existing scientific literature, with a main focus on the work by Nordbø and collaborators [7] and Espíndola and Blay [8]. Moreover, focus groups with patients and experienced psychiatrists and clinical psychologists were conducted at the University of Turin in order to ascertain both usefulness and reliability of the items included in this instrument. In fact, this collaborative effort yielded a self-report pilot questionnaire divided in three sections:
  I. General information on the course of AN;
  II. Investigation of three core areas (according to Espíndola and Blay [8]): a) triggers of the AN onset (7 items); b) meanings of AN (12 items); c) effects of AN (5 items);
  III. Identification of the most negatively affected area(s) of patients’ life.
    Clinical supervisors refined all questions and provided overall feedback to the research team. Given the need of generating an easy-to-administer tool, Visual Analogue Scales ranging from 0 (“strongly disagree”) to 10 (“strongly agree”) were adopted to score patients’ answers. VAS scales showed good reliability [27] and were chosen given their more robust metrical characteristics than discrete scales and their clinical utility in order to obtain unplanned responses.
- **Eating disorder inventory-2 (EDI-2** [28]**).** The EDI-2 is a self-report inventory that measures eating psychopathology through the evaluation of eating attitudes, behaviors and personality traits. Eleven subscales evaluate symptoms and psychological correlates of the eating disorders with high scores reflecting pathology.
- **Beck depression inventory (BDI** [29]**).** The BDI is a 13-item self-report questionnaire used to evaluate depressive symptoms according to the following scoring system: scores from 0 to 4 represent minimal depressive symptoms, scores of 5 to 7 indicate mild depression, scores of 8 to 15 indicate moderate depression and scores of 16 to 39 indicate severe depression.
- **Temperament and character inventory (TCI** [30]**).** The TCI is a 240-item self-administered questionnaire divided into 7 dimensions. Four of these dimensions assess temperament: novelty seeking (NS), harm avoidance (HA), reward dependence (RD), and persistence (P). The other three dimensions assess character: self-directedness (SD), cooperativeness (C), and self-transcendence (ST).
- **Anorexia Nervosa Stages of Change Questionnaire (ANSOCQ** [31]**).** The ANSOCQ is a 20-item self-report questionnaire designed according to the stages of change model of pre-contemplation, contemplation, preparation, action, and maintenance [32]. It evaluates a broad range of anorexic symptomatology including: eating behaviors, body shape and weight, emotional and interpersonal difficulties. Scores on each item of the ANSOCQ range from 1 (for the pre-contemplation-stage response) to 5 (for the maintenance-stage response).

### Statistical analysis

The Statistical Package for Social Sciences 21.0 (SPSS, SPSS Inc., Chicago, IL) was used for all analyses. A two-tailed alpha level of 0.05 was set.

Non parametric analyses have been used to compare diagnostic subtypes. After descriptive analyses, a principal component factor analysis (PCA) was performed on the items grouped in section II. We introduced the “meaning” items as quantitative variables. As we assumed factors were correlated, a rotation method was used. An R-matrix (Varimax rotation) was performed and multicollinearity was excluded. Kaiser–Meyer–Olkin measure of sampling was calculated to investigate sampling adequacy for conducting of factor analysis. Bartlett’s test of sphericity was also conducted. Factors was considered those with Eigenvalues > 1. Once factors were extracted, we assumed > 0.4 loading for a given variable to be significant.

Cronbach’s alpha has been calculated to measure the reliability of the three factors and of the 12-item section of the MANQ investigating the meanings of AN.

Bivariate correlations were run among factors, clinical variables, and results on the other questionnaires.

Multivariate regression analyses were carried out in order to ascertain which subscales of the MANQ were associated with clinical variables, eating psychopathology, and personality. Each factor derived by the factorial analysis was considered as a dependent variable. Clinical variables (Body Mass Index, age, duration of illness, age of onset, duration of treatment, duration of psychotherapy and number of hospitalizations), eating psychopathology (as measured by the EDI-2), and personality (as measured by the TCI) were considered independent variables and analyzed in three different blocks of regression.

## Results

### Participants’ clinical features

The sample was composed by 81 female patients diagnosed with AN; out of the total sample, 61 (75 %) were affected by the restricting (AN-R) and 20 (25 %) by the binge-purging (AN-BP) subtype. Please see Table 1 for socio-demographic and clinical variables.

**Table 1:** Clinical features of the sample

	AN patients (*n* = 81) Mean ± SD
BMI	15.1 ± 2.2
Ideal BMI	16.7 ± 2.0
Lowest BMI	13.3 ± 1.7
Age, years	25.3 ± 8.5
Age of onset, years	17.8 ± 4.2
Duration of illness, years	7.5 ± 7.8
Duration of outpatient treatment, months	21 ± 38.1
Number of delivered psychotherapy sessions	26.1 ± 48.5
Number of prior hospitalizations	1.9 ± 2.4

Legend: *BMI* Body Mass Index

### Meanings of AN

As shown in Table 2, according to the MANQ, the main factors triggering the onset of AN resulted to be: body dissatisfaction (6.6 ± 3.5), problems of adolescence (6.4 ± 3.3), and distress at school or work (5.4 ± 3.6) while not being able to identify a specific trigger scored poorly (2.5 ± 3.3).

**Table 2:** Patients’ scores on the Meanings of Anorexia Nervosa Questionnaire (MANQ) – Section II

Triggers of AN	Mean ± SD
Body dissatisfaction	6.6 ± 3.5
Problems of adolescence	6.4 ± 3.3
Distress at school/work	5.4 ± 3.6
Teasing about weight and body	5.2 ± 3.5
Separation, grief or loss of parents or other close family members	3.5 ± 3.9
No specific trigger	2.5 ± 3.3
Sexual harassment	1.5 ± 3.1
Meanings of AN	
Stability and safety	5.8 ± 3.7
Way to communicate	5.4 ± 3.5
Self-control	5.2 ± 3.7
Way to be valued and recognized	5.2 ± 3.9
Way to obtain affection and attention	5.0 ± 3.7
Way to feel beautiful	4.6 ± 4.0
Identity	4.4 ± 3.7
Power and control	4.3 ± 4.0
Avoidance of negative feelings and emotions	4.2 ± 3.7
Avoidance of negative experiences	4.1 ± 3.8
Way to die	3.9 ± 3.8
Illness denial	3.1 ± 3.4
Negative effects of AN	
Control	7.9 ± 3.0
Obsessions about body shape	6.9 ± 3.2
Loneliness	6.6 ± 3.4
Obsessions about food	6.3 ± 3.4
Counting calories	4.4 ± 3.7

Patients attributed to AN mostly the following meanings: AN as a source of balance and safety (5.4 ± 3.5) and as representing self-control ability (5.2 ± 3.7).

With respect to section III, the reported negative effects were mostly: being controlled by the illness (7.9 ± 3), obsessive thoughts about body shape (6.9 ± 3.2), and feeling alone (6.6 ± 3).

Moreover, 35.8 % of the sample reported on the MANQ the relations with peers and schoolmates as mostly impaired by AN, followed by health (29.9 %), family relationships (19.4 %), and school/work activities (16.4 %).

### Factor analysis of patients’ meanings of AN

Kaiser–Meyer–Olkin measure of sampling adequacy was 0.814 and Bartlett’s test of sphericity was significant (Chi-Square = 468.53, *p* < 0.0001) thus supporting the suitability of data for factor analysis.

Three Eigenvalues were greater than 1 which determined the number of factors computed. After Varimax rotation, three interpretable and clinically relevant factors were identified, capturing 65.33 % of the rotated variance. Table 3 shows the three factors and their item loadings with absolute values greater than 0.4 bolded. Factor 1, capturing 28.35 % of rotated variance, was labelled as “intrapsychic factor”, with positive significant loading for new identity, AN as not an illness, balance/safety, self-control, control/power, way to be valued and recognized, and attractiveness. Factor 2, capturing 20.67 % of rotated variance, represented a “relational factor” with positive significant loading for communication, way to be recognized, way to obtain affection and attention by others, and with negative significant loading with AN as not an illness. Factor 3, capturing 16.29 % of rotated variance, was named as “avoidance” and was defined by the avoidance of negative feelings, emotions, and experiences.

**Table 3:** Factor analysis of the Meanings of Anorexia Nervosa Questionnaire (MANQ)

	Factor 1 Intrapsychic	Factor 2 Relational	Factor 3 Avoidance
Identity	**0.512**	0.326	0.395
Stability and safety	**0.675**	0.330	0.294
Self-control	**0.789**	0.239	0.027
Power and control	**0.793**	0.318	−0.060
Way to feel beautiful	**0.707**	0.140	0.169
Illness denial	**0.654**	**−0.456**	0.181
Way to be valued and recognized	**0.520**	**0.632**	0.270
Way to obtain affection and attention	0.359	**0.833**	0.070
Way to communicate	0.264	**0.736**	0.249
Avoidance of negative emotions	0.172	0.069	**0.893**
Avoidance of negative experiences	0.084	0.331	**0.808**
Way to die	0.009	0.363	0.240

Loadings >0.4 are bolded

### Reliability and validity indicators

The Cronbach’s alpha of the 12 items on the meanings of AN was 0.869 while those of the factors 1, 2, and 3 were 0.853, 0.705, and 0.795, respectively.

Comparing AN-R and AN-BP individuals, a significant difference with respect to their ideal Body Mass Index (BMI) emerged (AN-R versus AN-BP: 17.1 ± 1.8 versus 15.4 ± 2, *p* = 0.003).

Concerning potential triggers, those with AN-BP scored significantly higher than AN-R on grief/separation (5.1 ± 4.4 versus 3 ± 3.6, *p* < 0.031) and sexual abuse (3.6 ± 4.3 versus 0.8 ± 2.3, *p* < 0.01). With respect to the meanings of AN, AN-BP individuals scored higher than AN-R on balance/safety (7.5 ± 2.8 versus 5.2 ± 3.9, *p* < 0.01) and on AN as a way to die (6 ± 3.8 versus 3.2 ± 3.6, *p* < 0.009). No other significant differences emerged between subtypes.

When compared to AN-R individuals, AN-BP patients were more depressed and showed higher NS and lower SD and C on the TCI. Moreover, AN-BP individuals reported greater bulimia, ineffectiveness, interoceptive awareness, asceticism, impulse regulation, and social insecurity on the EDI-2.

### Correlations between factors and clinical and psychometric variables

No correlations were found between factors and BMI, duration of illness, and duration of treatment/psychotherapy. Factor 2 “relational” negatively correlated with age of onset (*r* = −0.24, *p* < 0.05). Factor 3 “avoidance” positively correlated with age (*r* = 0.22, *p* < 0.05) and with the number of previous hospitalizations (*r* = 0.33, *p* < 0.003).

With respect to the other self-report questionnaires, Factor 1 (i.e., “intrapsychic factor”) positively correlated with the majority of the subscales on the EDI-2; on the TCI it positively correlated with HA (*r* = 0.28, *p* < 0.01) and negatively with SD (*r* = −0.37, *p* < 0.001). A negative correlation was also found between Factor 1 and the ANSOCQ score (*r* = −0.43, *p* < 0.001).

Factor 2 (i.e., “relational factor”) correlated positively with the majority of EDI-2 subscales and was negatively correlated to SD on the TCI (*r* = −0.38, *p* < 0.001).

Factor 3 (i.e., “avoidance”) was positively correlated with the majority of EDI-2 subscales and with HA on the TCI (*r* = 0.34, *p* < 0.002), while SD negatively correlated with this factor (*r* = −0.27, *p* = 0.05).

All factors positively correlated with the BDI score (see Table 4).

**Table 4:** Correlations between the three factors of the Meanings of Anorexia Nervosa Questionnaire (MANQ) and participants’ clinical data, eating psychopathology, and personality

	Factor 1 Intrapsychic	Factor 2 Relational	Factor 3 Avoidance
BMI	0.06	0.10	0.01
Age	−0.01	−0.02	0.22*
Age of onset	−0.06	−0.24*	0.11
Duration of illness	0.03	0.11	0.17
Duration of treatment, months	0.14	0.15	0.13
Duration of psychotherapy, number of sessions	−0.01	0.19	0.04
Number of prior hospitalizations	−0.01	0.08	0.33**
BDI	0.32**	0.34**	0.32**
ANSOCQ	−0.43**	−0.01	−0.07
EDI-2			
Drive for thinness	0.44**	0.25*	0.17
Bulimia	−0.09	0.29**	0.22*
Body dissatisfaction	0.22*	0.19	0.32**
Ineffectiveness	0.34**	0.28**	0.29**
Perfectionism	0.29**	0.22*	0.26**
Interpersonal distrust	0.28*	0.14	0.23*
Interoceptive awareness	0.23*	0.33**	0.35**
Maturity fears	0.21	0.28**	0.16*
Asceticism	0.41**	0.4**	0.22*
Impulse regulation	0.29**	0.45**	0.19
Social insecurity	0.24*	0.22*	0.3**
TCI			
Novelty seeking	0.01	0.1	−0.01
Harm avoidance	0.28**	0.2	0.34**
Reward dependence	−0.18	−0.01	−0.21
Persistence	0.14	0.01	−0.07
Self-directedness	−0.37**	−0.38**	−0.27*
Cooperativeness	−0.14	−0.19	−0.05
Self-transcendence	−0.14	0.05	−0.01

*p <0.05. **p < 0.01

Legend: *BMI* Body Mass Index, *BDI* Beck Depression Inventory, *EDI-2* Eating Disorders Inventory-2, *TCI* Temperament and Character Inventory, *ANSOCQ* Anorexia Nervosa Stages of Change Questionnaire

### Multivariate regression analysis of the association between factors and clinical variables, eating psychopathology, and personality

As shown in Table 5, no clinical variable was significantly associated with Factors 1 and 2 while number of hospitalizations was associated with Factor 3 (*p* < 0.001), even when controlling for all other clinical variables.

**Table 5:** Multivariate regression analyses between the three factors of the Meanings of Anorexia Nervosa Questionnaire (MANQ) and clinical variables, eating psychopathology, and personality

	Block 1^a^		Block 2^b^		Block 3^c^	
	F	p	F	p	F	p
Factor 1	0.927	0.491	3.529	**0.001**	2.115	0.052
Factor 2	1.259	0.282	2.719	**0.006**	2.144	**0.049**
Factor 3	2.833	**0.011**	1.542	0.137	2.323	**0.034**

^a^Regression analysis controlled for Body Mass Index, age, duration of illness, age of onset, duration of treatment, duration of psychotherapy and number of hospitalizations

^b^Regression analysis controlled for Eating Disorders Inventory-2 items

^c^Regression analysis controlled for Temperament and Character Inventory items

Significant *p*-values are bolded in the Table

With respect to EDI-2, drive for thinness was significantly associated with Factor 1 (*p* = 0.002), and impulse regulation with Factor 2 (*p* = 0.006); concerning the TCI, SD was significantly linked with Factors 1 (*p* = 0.044) and 2 (*p* = 0.025) and HA with Factor 3 (*p* = 0.027).

## Discussion

This study was conceived to present and use a novel instrument (MANQ) aimed to quantitatively analyze the potential triggers for AN, the meanings AN sufferers attribute to their illness, and the negative consequences of AN. With respect to the meanings of AN, our research identified three different factors which are related to patients’ personality and motivation to treatments but unrelated to duration of both illness and treatments. Taken together, our findings also confirmed previous literature, mostly with respect to triggers and AN-related negative effects [7, 8, 19].

With respect to the statistical analysis, the scale of the MANQ on the meanings of AN reported an overall solid statistic validity (Cronbach’s alpha = 0.853). Moreover, the section on meanings showed good validity indexes (i.e., capturing 65 % of the variance) when investigating the independent factors (i.e., intrapsychic, relational, and avoidance factors) underpinning the 12 questions of this section. Similarly, also the three identified factors resulted to be psychometrically robust (Cronbach’s alpha = 0.853, 0.705, and 0.795, for factors 1, 2, and 3, respectively). Therefore, this novel instrument may have clinical implications since it is not only statistically sound but also straightforward and easy to administer: in fact, only 5 min are required to complete all questions.

It is of note that the vast majority of patients could identify those elements that elicited the onset of their illness thus trying to offer an explanation for this moment of their lives. According to previous literature, patients were likely to report body dissatisfaction, teasing about weight and shape [33], and problems of adolescence [34] as potential triggers for the onset of AN. Still, those with a binge-purging subtype of AN reported significantly more often than those affected by a restricting subtype of AN sexual traumas, as expected given the existing literature on this topic [35, 36].

Similarly, we found data in line with previous literature also with respect to the subjective meanings of AN [8, 19]. For example, a broad consensus emerged about AN as a way to be protected or to communicate. It is noteworthy that AN-BP patients expressed significantly more frequently than those with AN-R that their eating disorder is a way to give up living; this is consistent on one hand with the data of the present paper on depressive symptoms reported by the two clinical subgroups and on the other hand with the existing body of evidence. In fact, those affected by the AN-BP subtype are known to be often plagued by severe psychopathology [37–39] and depression [40] and to describe their disorder as a source of safety more often than the AN-R group, potentially mirroring a worse prognosis [3, 41]. Longitudinal studies are worth assessing whether patients tend to score differently on this item in case of diagnostic cross-over or positive outcome. Also, it should be acknowledged that previous lines of research (i.e., P-CAN [21, 22], P-CED [23]) focused on the meanings of AN. Nevertheless, the MANQ could be used in the light of feasibility, given its easiness of administration (i.e., only 12 questions); still, this instrument pays close attention to the avoidance element providing also a broad range of possibilities of response (e.g., Likert-format responses). Moreover, this questionnaire captures also two different sections: the first one on triggering factors and the second one on the consequences of illness. Therefore, depending on the research questions, different instruments could be used in order to shed light on the multifaceted aspects of AN.

Being controlled by the disorder is most frequently reported as a negative consequence of AN, as already reported by our group [19]. Such an element can be useful in treatment: in fact, when patients realize to be overwhelmed and trapped by the disorder they somehow start to criticize it alike. Therefore, this may represent a good starting point in therapy and further research on this topic is recommended [42, 43].

Still, the factorial analysis conducted on patients’ meanings of AN provided interesting data. Three factors, explaining 65 % of the variance emerged and were labelled as intrapsychic, relational, and avoidance factors. Relatedly, a qualitative study conducted with recovered individuals reported how relevant can be overcoming the idea of AN as a way to perform control highlighting instead the need for improving relational skills and emotional management [44]. It is of interest that in our study these factors emerged as clearly separated: only the item assessing need for recognition was shared by both intrapsychic and relational factors, possibly because it could be referred to both personal and others’ recognition. Such an independence of the three factors could have clinical implications in the light of improving highly individualized treatments [10]. Accordingly, we found an inverse correlation between the relational factor and age of onset: in fact, it is well-known how both family- and communication-related elements can be relevant in treatment, particularly with adolescents [45]. Illness denial correlated with the intrapsychic and relational factors in a direct and inverse way, respectively. This finding is of interest since the intrapsychic factor seemed to be more related to the ego-syntonic aspects of AN while the relational factor would indicate greater awareness of illness, including the communication adaptive function of the disorder. Consistently, the intrapsychic factor inversely correlated with motivation to treatment; future studies are needed to investigate as to whether the relational factor (i.e., greater awareness of illness) could correlate with a favorable prognosis, even more so because ego-syntonicity and denial of illness are intertwined with resistance to treatments [1].

Avoidance was the most focused factor; however, it resulted to be independent of the intrapsychic one. A number of models described avoidance as an independent mechanism which is key, at least in a subgroup of patients, with respect to both onset and maintenance of AN [5, 6, 46–48]. The positive correlation between this factor and patients’ age and number of previous hospitalizations would require further investigations to ascertain whether avoidance can play a role in adulthood and in treatment planning.

All factors correlated with eating psychopathology as measured by the EDI-2 with no specific patterns of correlations. However, the greater the EDI-2 scores the greater the scores of the factors; this finding provides further support to the “adaptive function” of AN in the maintenance of the disorder [10]. Also, the more severe was the eating disorder the more patients seemed to value safety and structure somehow provided by AN [23]. Instead, when running multivariate regression analyses, the vast majority of correlations were not confirmed; however, the intrapsychic factor resulted associated with a core aspect of eating psychopathology, namely drive for thinness and the relational factor with impulse regulation.

With respect to personality, all factors inversely correlated with SD. Since low SD is suggestive of a fragile identity and difficulties with pursuing goals [30] this kind of correlation would raise the possibility that patients may need the disorder to help them shape their identity, as previously suggested [7, 8, 19]. Moreover, both intrapsychic and avoidance factors correlate with HA; this provides support to our a priori hypothesis, given the relevance of HA for AN patients [49–51] with multivariate regression analysis overall confirming this trend of association.

Finally, the last aim of this study was to determine the independence of subjective AN meanings from duration of both treatment and illness. The positive meanings of AN resulted to be independent of all other elements since no correlations were found with either duration of illness or treatment/psychotherapy. Therefore, the scarring effect of the illness could be excluded as well as an “over-rationalizing” effect of psychotherapy [9]. From a clinical standpoint this is encouraging since patients sometimes perceive to be “taught” what to feel and think in treatment, regrettably confirming their feelings of ineffectiveness and hindering a real awareness of their psychological resources [9].

This study suffers from some limitations: the questionnaire is not validated, the sample size of the AN-BP subgroup is modest, and to quantitatively measure patients’ opinions can be theoretically questioned. Nevertheless, our data are overall in line with qualitative studies on this topic and highlight some novel elements like the relationships between meanings of AN and illness severity and personality, independently of the treatment received.

## Conclusions

In closing, this study highlights that patients with AN frequently attribute a subjective meaning to their disorder and even more often tend to feel trapped and overwhelmed by the disorder itself. Such meanings correlate with both illness severity and patients’ character, independently of duration of illness and treatment. Sufferers’ perspectives and opinions on their disorder should be carefully considered in treatment because of their usefulness in both overcoming resistance [43] and engaging patients in treatment [52].

## Abbreviations

AN, anorexia nervosa; AN-BP, anorexia nervosa binge-purging subtype; AN-R, anorexia nervosa restricting subtype; ANSOCQ, Anorexia Nervosa Stages of Change Questionnaire; BDI, Beck Depression Inventory; BMI, body mass index; C, cooperativeness; DSM-IV-TR, Diagnostic and Statistical Manual of Mental Disorders – fourth edition – text revisions; EDI-2, Eating Disorders inventory-2; HA, harm avoidance; MANQ, Meaning of Anorexia Nervosa Questionnaire; NS, novelty seeking; SD, self-directedness; TCI, Temperament and Character Inventory.

## Additional file

Additional file 1: It is the full-text of the questionnaire made available for all researchers, clinicians and readers. (DOC 51 kb)
