# Supplementary material for: A factor analysis of the meanings of anorexia nervosa: intrapsychic, relational, and avoidant dimensions and their clinical correlates
Source: BMC Psychiatry. 2016 Jun 7;16:190. doi: 10.1186/s12888-016-0894-6 (PMC4897918; doi:10.1186/s12888-016-0894-6)
Supplement: Additional file 1: — It is the full-text of the questionnaire made available for all researchers, clinicians and readers. (DOC 51 kb) [file 12888_2016_894_MOESM1_ESM.doc]

**Meanings of Anorexia Nervosa Questionnaire (MANQ)**

LAST NAME: ____________________ FIRST NAME: ____________________

DATE OF BIRTH: ____________________

**Section I**

Age of onset of Anorexia Nervosa: _________

Current weight: _________ kg Current height: _________ cm

- What is your highest weight ever reached (excluding pregnancies)? _________

When did you reach this weight for the first time? _________

How long have you maintained this weight? _________

- What is your lowest weight ever reached (since you were 15 years old)? _________

When did you reach this weight for the first time? _________

How long have you maintained this weight? _________

- What is the weight you have maintained for the longest amount of time? _________

At what age did you reach this weight for the first time? _________

- In your opinion, what is your ideal weight? _________
- After being diagnosed with Anorexia Nervosa, how long have you been receiving outpatient treatment? _________

How many months have you been receiving psychotherapy? _________

- After being diagnosed with Anorexia Nervosa, have you been fully or partially hospitalized? _________

If so, how many times? _________

**Section II**

Please place a cross on the scale below indicating the point that best matches your agreement or disagreement.

0= Strongly disagree

100= Strongly agree

Example: 0_____________________________________100

1. **FACTORS THAT COULD HAVE TRIGGERED THE ONSET OF ANOREXIA NERVOSA**

In my opinion, the onset of my symptoms is attributable to:

1. Distress at school or work

0_____________________________________100

1. Teasing about weight and body

0_____________________________________100

1. Separation, grief or loss of parents or other close family members

0_____________________________________100

1. Body dissatisfaction

0_____________________________________100

1. Problems of adolescence (peers, puberty, emotional distress, etc.)

0_____________________________________100

1. Sexual harassment

0_____________________________________100

1. No specific event

0_____________________________________100

**B) MEANING ATTRIBUTED TO ANOREXIA NERVOSA**

1. I often thought that anorexia nervosa represents a new identity which is preferable than the previous one.

0_____________________________________100

1. I think anorexia nervosa is a way to avoid negative feelings and emotions.

0_____________________________________100

1. I think anorexia nervosa is a way to avoid unpleasant experiences through the focus on weight and body; as a result, I do not have other energy to face difficulties or practical problems.

0_____________________________________100

1. I often think anorexia nervosa is not an illness.

0_____________________________________100

1. It seems to me anorexia nervosa is a way to communicate my difficulties to my family members.

0_____________________________________100

1. My illness is a source of stability and safety since my life is ordered by rules and rituals mostly regarding eating, weight, and body.

0_____________________________________100

1. I think that anorexia nervosa represents my self-control ability.

0_____________________________________100

1. I often think that anorexia nervosa is a way to achieve control and power.

0_____________________________________100

1. I often associate anorexia nervosa with a way to be valued and recognized by others.

0_____________________________________100

1. I often think this is a way to obtain affection and attention by others.

0_____________________________________100

1. I frequently think of underweight as a way to feel beautiful and attractive.

0_____________________________________100

1. I think anorexia nervosa is an informed way to leave me dying.

0_____________________________________100

**C) EFFECTS OF ANOREXIA NERVOSA**

1. It is vital for me to calculate the calories of foods before eating and sometimes I feel my head is full of numbers

0_____________________________________100

1. I often think about food which is now my main interest.

0_____________________________________100

1. I frequently think about my body shape.

0_____________________________________100

1. I have often felt alone because of anorexia nervosa.

0_____________________________________100

1. I often think that anorexia nervosa controls and traps me.

0_____________________________________100

**Section III**

In your opinion what is the area in your life that has been mostly damaged by anorexia nervosa? Please select one answer:

- 1. School/work activities
  2. Relations with peers and schoolmates
  3. Family relationships
  4. Health
  5. Other (please specify)__________________________________________

Thank you for taking this survey.
